# Supplementary material for: The cytochrome b carboxyl terminal region is necessary for mitochondrial complex III assembly
Source: Life Sci Alliance. 2023 Apr 24;6(7):e202201858. doi: 10.26508/lsa.202201858 (PMC10132202; doi:10.26508/lsa.202201858)
Supplement: Supplementary file 1 [file LSA-2022-01858_TableS1.docx]

**Supplemental Table S1. Yeast strains used in the present study.**

| **Strain** | **Nuclear (mitochodrial) genotype** | **Reference/source** |
| --- | --- | --- |
| NAB69 | *Mata,ade2-101, arg8-delta::hisG, ura3-52, kar1-1 (ρ^0^)* | (Bonnefoy *et al*, 2001) |
| NB71 | *Matα*, *ade2-101, ura3-52, leu2-delta, arg8-delta::URA3, kar1-1, ( cox3Δ:ARG8^m^-1)* | (Bonnefoy *et al.*, 2001) |
| BY4742 | *Matα, his3-delta1, leu2-delta0, lys2-delta0, ura3-delta0, BY4742* | YKO Mat*α* Strain Collection (Open Biosystems) |
| NB40-36a | *Matα*, *arg8::hisG, leu2-3,112, lys2, ura3-52, D273-10B* | (Perez-Martinez *et al.*, 2003) |
| DFM1 | *Mata*, *ade2-101, arg8-delta::hisG, ura3-52, kar1-1 ^a^(ρ^-^, pYCV66)* | This study |
| DFM2 | *Matα*, *arg8::URA3, his3-delta1, leu2-delta0, lys2-delta0, ura3-delta0, BY4742 ( ^b^ ΔΣ ai, cobΔ::ARG8^m^)* | (García-Guerrero *et al.*, 2018) |
| DFM4 | *Mata*, *ade2-101, arg8-delta::hisG, ura3-52, kar1-1 (ρ^-^, pMD2)* | This study |
| DFM6 | *Matα*, *arg8::URA3, his3-delta1, leu2-delta0, lys2-delta0, ura3-delta0, BY4742 (ΔΣ ai, ^c^ΔΣ bi)* | This study |
| DFM7 | *Mata*, *ade2-101, arg8-delta::hisG, ura3-52, kar1-1 (ρ^-^, pDM4)* | This study |
| DFM8 | *Mata*, *ade2-101, arg8-delta::hisG, ura3-52, kar1-1(ρ^-^, pDM6)* | This study |
| DFM9 | *Matα*, *arg8::URA3, his3-delta1, leu2-delta0, lys2-delta0, ura3-delta0, BY4742 (ΔΣ ai, COBΔC13)* | This study |
| DFM10 | *Matα*, *arg8::URA3, his3-delta1, leu2-delta0, lys2-delta0, ura3-delta0, BY4742 (ΔΣ ai, COBΔC8)* | This study |
| DFM11 | *Matα*, *arg8::URA3, his3-delta1, leu2-delta0, lys2-delta0, ura3-delta0, cbp3Δ::KANMX4, BY4742 (ΔΣ ai, COBΔC8)* | This study |
| DFM12 | *Matα*, *arg8::URA3, his3-delta1, leu2-delta0, lys2-delta0, ura3-delta0, cbp6Δ::LEU2, BY4742 (ΔΣ ai, COBΔC8)* | This study |
| DFM13 | *Matα*, *arg8::URA3, his3-delta1, leu2-delta0, lys2-delta0, ura3-delta0, qcr7Δ::KANMX4, BY4742 (ΔΣ ai, COBΔC8)* | This study |
| DFM14 | *Matα*, *arg8::URA3, his3-delta1, leu2-delta0, lys2-delta0, ura3-delta0, cbs1Δ::HIS3, BY4742 (ΔΣ ai, COBΔC8)* | This study |
| DFM15 | *Mata*, *ade2-101, arg8-delta::hisG, ura3-52, kar1-1 (cobΔ::ARG8^m^)* | This study |
| DFM16 | *Matα*, *arg8::hisG, leu2-3,112, lys2, ura3-52, D273-10B (ΔΣ ai, cobΔ::ARG8^m^)* | This study |
| DFM21 | *Matα*, *arg8::hisG, leu2-3,112, lys2, ura3-52, D273-10B (ΔΣ ai, ΔΣ bi)* | This study |
| DFM24 | *Matα*, *arg8::hisG, leu2-3,112, lys2, ura3-52, D273-10B (ΔΣ ai, COBΔC13)* | This study |
| DFM25 | *Matα, his3-delta1, leu2-delta0, lys2-delta0, ura3-delta0, cbp3Δ::KANMX4, ^d^HIS3::pBpa-RS::tRNA^CUA (pBpa)^*, *BY4742* | This study |
| DFM26 | *Matα*, *arg8::URA3, his3-delta1, leu2-delta0, lys2-delta0, ura3-delta0, cbp3Δ::KANMX4, HIS3::pBpa-RS::tRNA^CUA (pBpa)^*, *BY4742 (ΔΣ ai, COBΔC13)* | This study |
| AGG26 | *Matα*, *arg8::URA3, his3-delta1, leu2-delta0, lys2-delta0, ura3-delta0, BY4742 (ΔΣ ai)* | (García-Guerrero *et al.*, 2018) |
| AGG58 | *Matα, his3-delta1, leu2-delta0, lys2-delta0, ura3-delta0, cbp3Δ::KANMX4, BY4742* | (García-Guerrero *et al.*, 2018) |
| AGG59 | *Matα, his3-delta1, leu2-delta0, lys2-delta0, ura3-delta0, cbp6Δ::LEU2, BY4742* | (García-Guerrero *et al.*, 2018) |
| AGG68 | *Mata, his3-delta1, leu2-delta0, met15-delta0, ura3-delta0, qcr7Δ::KANMX4, BY4742* | (García-Guerrero *et al.*, 2018) |
| AGG77 | *Matα, his3-delta1, leu2-delta0, lys2-delta0, ura3-delta0, cbs1Δ::URA3, BY4742* | (García-Guerrero *et al.*, 2018) |
| AGG78 | *Matα*, *arg8::hisG, leu2-3,112, lys2, ura3-52, cbs1Δ::URA3, D273-10B* | (García-Guerrero *et al.*, 2018) |
| YCV167 | *Matα*, *arg8::URA3, his3-delta1, leu2-delta0, lys2-delta0, ura3-delta0, qcr7Δ::KANMX4, BY4742 (ΔΣ ai, COBΔC13)* | This study |
| YCV168 | *Matα*, *arg8::URA3, his3-delta1, leu2-delta0, lys2-delta0, ura3-delta0, cbs1Δ::HIS3, BY4742 (ΔΣ ai, COBΔC13)* | This study |
| YCV169 | *Matα*, *arg8::URA3, his3-delta1, leu2-delta0, lys2-delta0, ura3-delta0, cbp3Δ::KANMX4, BY4742 (ΔΣ ai, COBDC13)* | This study |
| YCV170 | *Matα*, *arg8::URA3, his3-delta1, leu2-delta0, lys2-delta0, ura3-delta0, cbp6Δ::LEU2, BY4742 (ΔΣ ai, COBΔC13)* | This study |
| YVC171 | *Matα*, *arg8::URA3, his3-delta1, leu2-delta0, lys2-delta0, ura3-delta0, cbs1Δ::HIS3, BY4742 (ΔΣ ai, ΔΣ bi)* | This study |
| YCV176 | *Matα*, *arg8::URA3, his3-delta1, leu2-delta0, lys2-delta0, ura3-delta0, cbp3Δ::KANMX4, BY4742 ΔDΣ ai, ΔΣ bi)* | This study |
| YCV177 | *Matα*, *arg8::URA3, his3-delta1, leu2-delta0, lys2-delta0, ura3-delta0, qcr7Δ::KANMX4, BY4742 (ΔΣ ai, ΔΣ bi)* | This study |
| YCV178 | *Matα*, *arg8::URA3, his3-delta1, leu2-delta0, lys2-delta0, ura3-delta0, cbp6Δ::LEU2, BY4742 (ΔΣ ai, ΔΣ bi)* | This study |
| M17-162 | *Matα*, *met6 (cob2)* | (Bonjardim *et al.*, 1996) |
| M17-162 4D | *Mata*, *ade1 (cob2)* | (Bonjardim *et al.*, 1996) |

*^a^* Parenthesis refer to the mitochondrial genotypes

*^b^ΔΣ ai* refers to the intronless *COX1* gene

*^c^ΔΣ bi* refers to the intronless *COB* gene

*^d^ pBpa-RS* refers to the tRNA synthetase for pBpa and *tRNA^CUA (pBpa)^*refers to the tRNA for pBPa
